# Sleep dysfunction mediates the relationship between hemodynamic response height of the secondary somatosensory cortex and fear-somatic symptom severity bidirectionally in young adults

**DOI:** 10.21203/rs.3.rs-10441569/v1

**Published:** 2026-09-02

**Authors:** Dandan Zhong, Lin Yang, Ning Zhang, Zhao Zhang, Yan Yan, Sihan Niu, Jianyu Dong, Yiyao Yelin, Ying Li, Guangfei Li, Chiang-Shan R. Li

**Affiliations:** Beijing University of Technology; Beijing University of Technology; Capital Medical University; Beijing University of Technology; Hebei Medical University; Capital Medical University; Capital Medical University; Universitat Politècnica de València; Beijing University of Technology; Beijing University of Technology; Yale University

**Keywords:** insomnia, brain imaging, hemodynamic response function, secondary somatosensory cortex, emotion, fear-somatic

## Abstract

**Background:**

Sleep dysfunction exerts profound effects on cognition and emotion. Among neurophysiological indices, the hemodynamic response height (HRH), as derived from functional magnetic resonance imaging (fMRI), represents a key marker of neural activities. Here, we addressed the interplay amongst sleep quality, HRH, and negative emotional states in young adults.

**Methods:**

We curated fMRI and clinical data of 735 young adults (373 women) from the Human Connectome Project. All participants were assessed for sleep quality with the Pittsburgh Sleep Quality Index (PSQI) and subjective emotional state with the NIH Toolbox of Negative Affect. Imaging data were processed with published routines and evaluated with a corrected threshold. In whole-brain regression we identified regional HRH correlates of PSQI score and, in mediation analysis, assessed the interrelationship amongst the regional HRH, PSQI, and clinical measures.

**Results:**

With age and sex as covariates, lower HRH in the secondary somatosensory cortex (SII) was associated with higher PSQI score across all subjects. No clusters were identified for hemodynamic time to peak or full width at half maximum at the same threshold. Both SII HRH and PSQI score were significantly correlated with fear-somatic symptom severity. Moreover, mediation analyses showed that PSQI score mediated the relationship between SII HRH and fear-somatic score bidirectionally.

**Conclusions:**

Reduced hemodynamic responsiveness in the SII is associated with poorer sleep quality and heightened somatic fear symptoms. These findings suggest that sleep dysfunction mediates a bidirectional link between somatosensory activities and emotional dysregulation, highlighting a potential mechanism of affective dysfunction in link with sleep deficiency.

## 1. Introduction

### 1.1 Impact of sleep deficiency on brain health

Sleep deficiency, resulting from suboptimal sleep duration or quality, is highly prevalent in modern society (Gohari et al., 2022). Sleep deficiency incurs cellular stress and damage and poses a risk to brain health (Coulson et al., 2022), including accelerated deposition of β amyloid and tau proteins in the brain (Lv et al., 2022). Sleep deficiency also disrupts cognitive functions such as attention, memory, and executive functions (Li et al., 2024a; Li et al., 2021; Li et al., 2024c; Liu et al., 2024; Ochab et al., 2021), likely by compromising structural and functional integrity of frontal brain regions (Belluardo et al., 2025; Robbins et al., 2021). Chronic insomnia can lead to long-lasting changes in grey matter volumes of the brain (Li et al., 2024d). Developmentally, excessively long sleep duration in middle childhood and short sleep in adolescence are both associated with behavioral problems during adolescence (Zhu et al., 2024).

### 1.2 Impact of sleep deficiency on emotion

Sleep deficiency exerts substantial adverse effects on emotion and may be involved in the pathophysiology of anxiety and depression (Nelson et al., 2022; Palmer et al., 2024). Epidemiological studies show that sleep disturbances affect nearly 50% of individuals with an anxiety disorder (Chellappa and Aeschbach, 2022). Patients with poor sleep quality exhibit higher rates of depression, when compared to those with adequate sleep (Hadlandsmyth et al., 2020; He and Ruan, 2022). Findings from the U.S. National Health and Nutrition Examination Survey revealed a dose-response relationship between sleep duration and depression, with both short (< 7 hours) and long (> 9 hours) sleep durations associated with elevated risk of clinically relevant depression (Chunnan et al., 2022).

Significantly correlated with psychological stress and somatic pain (Mancone et al., 2024), sleep deficiency has also been shown to partially mediate the association between cognitive decline and depressive symptoms (Liu et al., 2022). Sleep deficiency is significantly associated with negative somatic states, with symptoms such as shortness of breath, nausea, dizziness, muscle soreness, and pain in multiple body parts (Carrillo-Gonzalez et al., 2021; Ikeda et al., 2024; Jones et al., 2022; Yin and Zhang, 2024). Conversely, emotional factors, including fear, social alienation, depressive and somatic symptoms are predictive of sleep deficiency (Lelisho et al., 2023), suggesting bidirectional interplay between sleep deficiency and negative emotion and related problems. Emotion dysregulation mediated the relationship between sleep deficiency and stress, anger, hostility, and aggression (Demichelis et al., 2023), suggesting the impact of sleep dysfunction on emotional regulation and, in turn, mal-adaptive behaviors. However, the neural mechanisms underlying the impact of sleep deficiency on negative emotional states have not been investigated in the literature.

### 1.3 Hemodynamic response height as a neural measure

Functional magnetic resonance imaging (fMRI) is a non-invasive tool to characterize structural and functional brain changes. In fMRI, the hemodynamic response function (HRF) describes local changes in cerebral hemodynamics and oxygen metabolism due to briefly evoked neural activity (Fesharaki et al., 2024). Neural activity affects the timing and shape of hemodynamic response via changes in cerebral blood flow (CBF) and neurovascular coupling (Rangaprakash et al., 2021). The HRF is typically characterized by three main parameters – hemodynamic response height (HRH), time to peak (TTP), and full width at half maximum (FWHM), which represent the amplitude, delay, and duration, respectively, of the HRF.

These HRF parameters have been used as indicators of cerebral dysfunction (Fesharaki et al., 2024). For instance, West and colleagues reported increased TTP and reduced HRH in the auditory, visual, and motor cortices of older as compared to younger individuals (West et al., 2019). Aberrant HRF profiles were noted in obsessive compulsive disorder, which normalized following cognitive behavioral therapy, and pre-treatment HRH in the caudate predicted symptom severity post-treatment (Rangaprakash et al., 2021). HRF abnormalities have been identified in the precuneus of individuals with autism spectrum disorder (Yan et al., 2018). Recent findings by Bravi et al. demonstrated that variations in HRF shape, including reduced TTP, and elevated fractional amplitude of low frequency fluctuation in the default mode network, are associated with kynurenine metabolism, suggesting a vascular mechanism through which metabolic changes may influence brain function (Bravi et al., 2025). On the other hand, no studies to our knowledge have utilized HRH as a neural index to investigate the impact of sleep deficiency on brain function and behavioral health.

### 1.4 The Present Study

Here, we investigated whether and how sleep deficiency and negative emotions are related to regional hemodynamic responses of the brain. We curated the Human Connectome Project (HCP) data and employed a regression analysis of hemodynamic parameters against the Pittsburgh Sleep Quality Index (PSQI) score – an index of sleep quality – in a cohort of over 700 participants. To anticipate, we identified HRH correlates of emotional problems in the secondary somatosensory cortex (SII) and tested the inter-relationship among SII HRH, PSQI score, and emotional measures. Finally, we performed mediation analysis to characterize the interrelationships among PSQI scores, SII HRH, and emotional measures.

## 2. Materials and Methods

### 2.1 Dataset and demographics

We employed the HCP 1200 Subjects Release (S1200) data, collected from 2012 to 2015, in the current study. A total of 1096 young adults completed a resting state fMRI scan and, after exclusion of subjects missing physiological data (n = 80) or not meeting the scrubbing criteria (n = 146; details in “*2.3 Imaging protocol and preprocessing*”), 870 were retained. Hemodynamic response function (HRF) parameters were extracted from these 870 participants. Among them, 135 individuals with extensive regional signal loss or severe imaging artifacts were excluded from subsequent analyses, resulting in a final sample of 735 participants for further analysis. All subjects were physically healthy with no neurodevelopmental, neuropsychiatric or neurological disorders. Age and sex were included as covariates in the analyses of all subjects. The HCP study was approved by the Washington University Institutional Review Board (IRB #201204036).

### 2.2 Clinical and cognitive measures

All participants were assessed with the NIH Negative Affect Toolbox Survey on six negative emotions, including anger-affect, anger-hostility, anger-physical aggression, fear-affect, fear-somatic arousal, and sadness. Among them, fear-somatic arousal assesses somatic symptoms of anxiety that reflect autonomic arousal and perceptions of threat, using a 6-item fixed-length form (**Supplementary Methods**). Higher Fear-Somatic scores are indicative of more fear-somatic arousal.

Participants were evaluated with the Pittsburgh Sleep Quality Index (Buysse et al., 1989), which contains 19 self-rated questions. The 19 self-rated items are combined to 7 component scores, each of which ranged from 0 to 3, with “0” and “3” each indicating no and severe difficulty with sleep, respectively. Thus, individuals ranged from 0 to 21 in PSQI score, with a higher score representing worse sleep quality.

Table 1 shows age, years of education, PSQI, Fear-Somatic scores of men and women separately.

### 2.3 Imaging protocol and preprocessing

Imaging protocols and data preprocessing routines are described in **Supplementary Methods** (Li et al., 2023a; Li et al., 2024b; Li et al., 2023b). Participants completed 7 task and 2 resting-state (rs) scans on two separate days. Within each rs-fMRI scan, oblique axial acquisitions alternated between phase encoding in a right-to-left (RL) direction in one run and left-to-right (LR) direction in the other run. Each run lasted 14.4 min (1200 frames). Cardiac and respiratory data were also acquired, using a standard Siemens pulse oximeter placed on a digit and a respiratory belt placed on the abdomen. These physiological signals were sampled at 400 Hz (~ 288 samples per frame). More details of the data collection procedures can be found in the HCP S1200 Release Reference Manual.

### 2.4 Deconvolution of BOLD signal and estimation of the HRF

Hemodynamic response function (HRF) represents a mathematical transfer function – a model of the BOLD signal based on corresponding local neural activity (Fig. 1A). We used the MATLAB toolkit *rsHRF* to perform deconvolution and obtain HRF parameters, including HRH, TTP, and FWHM for the whole brain (Wu et al., 2021).

In group analyses, a whole-brain regression of HRF parameters against PSQI score was conducted in the entire sample with age and sex as covariates. The results were evaluated at voxel p < 0.001, uncorrected, in combination with a cluster p < 0.05, corrected for family-wise error (FWE) of multiple comparisons, on the basis of Gaussian random field theory, as implemented in SPM (Poldrack et al., 2008). For the regions of interest (ROIs) identified from the regression, we used the *REST* toolkit (Song et al., 2011) to derive for individual subjects the β estimates.

### 2.5 Mediation analyses

We performed mediation analyses following published routines (MacKinnon et al., 2007; Wager et al., 2008), as detailed earlier (Hu et al., 2018; Ide et al., 2017; Zhornitsky et al., 2019) (**Supplementary Methods**), to evaluate the relationships among regional HRF parameters, negative emotional measures and PSQI scores. The results of mediation analyses did not imply causality but served to clarify the inter-relationships of multiple correlating variables.

## 3. Results

### 3.1 Clinical measures

PSQI (*t* = −0.99, *p* = 0.322) and Fear-Somatic (*t* = −1.28, *p* = 0.202) scores showed no significant difference between men and women. **Supplementary Table S1** shows the results on tests of sex differences in other negative emotional measures.

PSQI score showed a significant correlation with Fear-Somatic score (all: *r* = 0.22, *p* < 0.001; men: *r* = 0.21, *p* < 0.001; women: *r* = 0.22, *p* < 0.001). In a slope test men and women did not differ in the slope of regression of PSQI and Fear-Somatic scores (*Z* = −0.17, *p* = 0.865; Fig. 2A). Note that these correlations were significant even with correction for multiple comparisons. **Supplementary Table S2** shows the results of correlation of PSQI score and other negative emotional measures.

### 3.2 HRF Parameters correlate with PSQI

Whole-brain linear regression of the HRH for the entire sample revealed a significant negative correlation with PSQI score in a single cluster in the left Rolandic operculum (x, y, z = −46, −12, 18; peak T = 4.35, cluster size (k) = 195, cluster p < 0.001; Fig. 1B), with the bulk of voxels located in the secondary somatosensory cortex (SII; Fig. 1C). Whole-brain linear regression of TTP or FWHM against PSQI score did not reveal any clusters at the same threshold.

The *β* estimate of the SII HRH was significantly correlated with PSQI score across all subjects, as expected (r = −0.149, p < 0.001). When examined separately for men and women, the correlations were also both significant (men: r = −0.109, p = 0.038; women: r = −0.178, p = 0.001). In a slope test men and women did not differ in the slope of regression (Z = 0.95, p = 0.342; Fig. 2B).

We next examined whether the *β* estimates of SII HRH were significantly correlated with Fear-Somatic scores. The results showed a significant correlation across all subjects (*r* = −0.109, *p* = 0.003) and in women (*r* = −0.121, *p* = 0.019), but not in men (*r* = −0.094, *p* = 0.076) alone. However, in a slope test men and women did not differ in the slope of regression (*Z* = 0.37, *p* = 0.711; Fig. 2C). **Supplementary Table S3** shows the results of correlation of SII HRH and other negative affect measures, and none of other negative emotion metrics were significantly associated with SII HRH. Note that the correlation of SII HRH with Fear-Somatic score across all subjects (p = 0.003) survived correction for multiple comparisons (at p < 0.05/6 = 0.008) even when we considered all six emotional measures in the analyses.

### 3.3 Inter-relationship of SII HRH, PSQI, and Fear-Somatic scores

In the entire sample, individual PSQI score, Fear-Somatic, and SII HRH were significantly correlated pairwise. We performed a mediation analysis to examine the inter-relationship amongst the PSQI, Fear-Somatic score, and SII HRH, with age and sex as covariates. We considered all six models and employed a corrected *p* (0.05/6 = 0.0083) to evaluate the mediation effects. Two models (SII HRH → PSQI score → Fear-Somatic scores; Fear-Somatic scores → PSQI score → SII HRH) showed significant and complete mediation (Fig. 3; Table 2).

## Discussion

We demonstrated that sleep deficiency, as reflected in higher PSQI score, was associated with lower HRH in SII, a hub for processing and integrating somatosensory information. PSQI and Fear-Somatic scores were both negatively correlated with the SII HRH, whereas a positive correlation was found between PSQI and Fear-Somatic scores. Further, sleep deficiency bidirectionally mediated the relationship between SII HRH and fear-somatic arousal. Together, these findings suggest a role of sleep deficit in inter-linking altered somatosensory signaling and fear-somatic arousal and provide systems level evidence for the impact of dysfunctional sleep on emotion in young adults.

### 4.1 Sleep deficiency and reduced hemodynamic response in SII

Within the parietal operculum, SII is located in the depths of the lateral sulcus and positioned between the primary somatosensory cortex (SI) and insular cortex (Zlatkina et al., 2022). Previous studies have associated structural, functional and hemodynamic abnormalities of the somatosensory cortex with sleep deficiency. For instance, sleep deprivation (SD) affects somatosensory cortical excitability, and higher level of cortical responsiveness after sleep loss may account for post-SD alterations in vigilance and behavior (Gorgoni et al., 2014). Lou et al. identified a noradrenergic pathway from the locus coeruleus to the hindlimb SI in mice, through which SD induced pain hypersensitivity (Lou et al., 2024). This finding suggests a role of the somatosensory cortex in mediating adverse sensory and perceptual consequences of sleep disruption. More broadly from the clinical literature, the increase in functional connection between bilateral somatosensory cortex and thalamus is negatively correlated with sleep spindle density, a key electrophysiological indicator of sleep quality and brain health, in schizophrenia (Baran et al., 2019). Patients with restless legs syndrome (RLS) experience sleep problems and, relative to healthy controls, showed morphological changes in the somatosensory system (Lee et al., 2018; Unrath et al., 2008). Stimulating the left SI alleviated subjective symptom severity in patients with RLS (Lanza et al., 2023).

Sleep deficiency affects cerebral blood flow (CBF) and hemodynamic responses of multiple brain regions, including the SII. For instance, SD for 24 hours led to cognitive impairments, altered cortical CBF and neurovascular coupling (Csipo et al., 2021). Compared to controls, patients with isolated rapid-eye-movement sleep behavior disorder exhibited profound cortical hypoperfusion and microvascular flow disturbances across the cortex (Eskildsen et al., 2021). Similarly, male patients with obstructive sleep apnea showed reductions in overall and regional CBF and cerebral blood volume relative to healthy controls (Yan et al., 2021). Regional hypoperfusion across a wide swath of brain areas, including those in the parietal cortex, was notably correlated with intermittent hypoxia and sleep fragmentation (SF) (Yan et al., 2021). In mice, chronic intermittent hypoxia, as often occurs during SD, impeded neurovascular coupling (Capone et al., 2012). In mice under chronic SF relative to control mice, the cerebrovascular network density increased, suggesting altered vascular dynamics, in the cerebral cortex, hippocampus, and striatum. Furthermore, SF significantly dampened the increase in CBF elicited by whisker stimulation in anesthetized mice (Xue et al., 2023). HRF describes the speed and pattern of changes in local CBF and BOLD signals triggered by neuronal activity. The lower HRH associated with poorer sleep quality may thus suggest insufficient blood supply to the SII area.

### 4.2 SII and fear-somatic negative emotion

Functionally, the SII bridges sensation, cognition, and emotion (Yang et al., 2025), playing a core role in tactile object recognition (Ohte et al., 2025; Rossi-Pool et al., 2021), integration of bilateral sensory information (Guran et al., 2024; Pala and Stanley, 2022), as well as pain and emotion processing (Huang et al., 2025; Riva et al., 2022; Shraim et al., 2024). In a study of healthy young adults exposed to negative, neutral, or positive valence images from the International Affective Picture System, as well as laser pain stimulation, the interaction between negative emotions and laser stimulation was associated with left SII activation (Orenius et al., 2017). Among women with chronic postoperative pain, activation of the left somatosensory cortex was positively correlated with depression and anxiety symptom severity (Moreland et al., 2025). Greater depressive mood was associated with stronger regional (SII, posterior insula) responses that support pain assessment and sensation discrimination and with higher individual pain sensitivity (Adler-Neal et al., 2019). State anxiety was positively correlated with the amplitude of low-frequency fluctuations (ALFFs) of somatomotor regions, including the right postcentral and precentral gyri (Li et al., 2019). Individuals with high trait anxiety, who are more sensitive to somatosensory and interoceptive signals, e.g., muscle tension and changes in heart rate, showed stronger somatosensory and insular cortical activation in a fear generalization task (Huggins et al., 2021).

Notably, somatic symptoms caused by anxiety or fear, including palpitations, sweating and muscle tension are “felt” because of changes in anterior insula-somatosensory-motor network activities (Tamietto et al., 2015). Chronic sleep deficiency can lead to altered mood, greater anxiety, elevated physiological arousal, and somatic symptoms (Dauvilliers et al., 2025; Dumser et al., 2023; Gold et al., 2016; Seton and Fitzgerald, 2021; Short et al., 2022). In turn, excessive arousal, negative emotions, and somatic symptoms contribute to the development and chronicity of insomnia (Gorgoni et al., 2021; Muehlan et al., 2023). In patients with sleep disordered breathing, there is a strong association between the level of somatic arousal and the presence of stress-related, including anxiety and sleep disorders (Amdo et al., 2016). Furthermore, somatic symptoms, including loss of appetite and sleep disturbances, were observed in people with parietal cortical lesions that encompassed the SII (Krick et al., 2023). Finally, somatic symptom severity was associated with reduction in gray matter volumes of the SII (Wei et al., 2020). Together, the current findings along with this literature support that SII and fear-somatic negative emotions were closely related.

### 4.3 Limitations of the study and conclusions

A number of limitations need to be considered. First, fMRI provides an indirect measure of neural activity (Rangaprakash et al., 2017). The HRF obtained through deconvolution of the BOLD signals is not a direct measurement of CBF either. In future studies, HRF can be characterized along with indicators of direct measurement of CBF, with e.g., arterial spin labeling imaging. Second, we focused on resting-state data in analysis. Studies with behavioral paradigms of negative emotion processing and concurrent skin conductance recording may provide behavioral, physiological, and imaging data to investigate how sleep deficiency alters arousal and affective behavior as well as the neural bases of these effects. Finally, although sleep quality and negative emotions were quantified respectively using the PSQI and NIH Negative Affect Toolbox Survey, the HCP participants were largely healthy, with those meeting clinical diagnoses and/or requiring treatment excluded from enrollment. Thus, whether or how the current findings may extend to clinical populations remain to be seen.

To conclude, resting-state SII HRH is diminished in young adults with more severe sleep problems. Sleep dysfunction inter-link lower SII HRH and higher severity of fear-somatic arousal bidirectionally.

## Supplementary Material

Supplementary Files

This is a list of supplementary files associated with this preprint. Click to download.

- SupplementHRFheightPSQIfearsomatic.docx

## Figures and Tables

**Figure 1:**
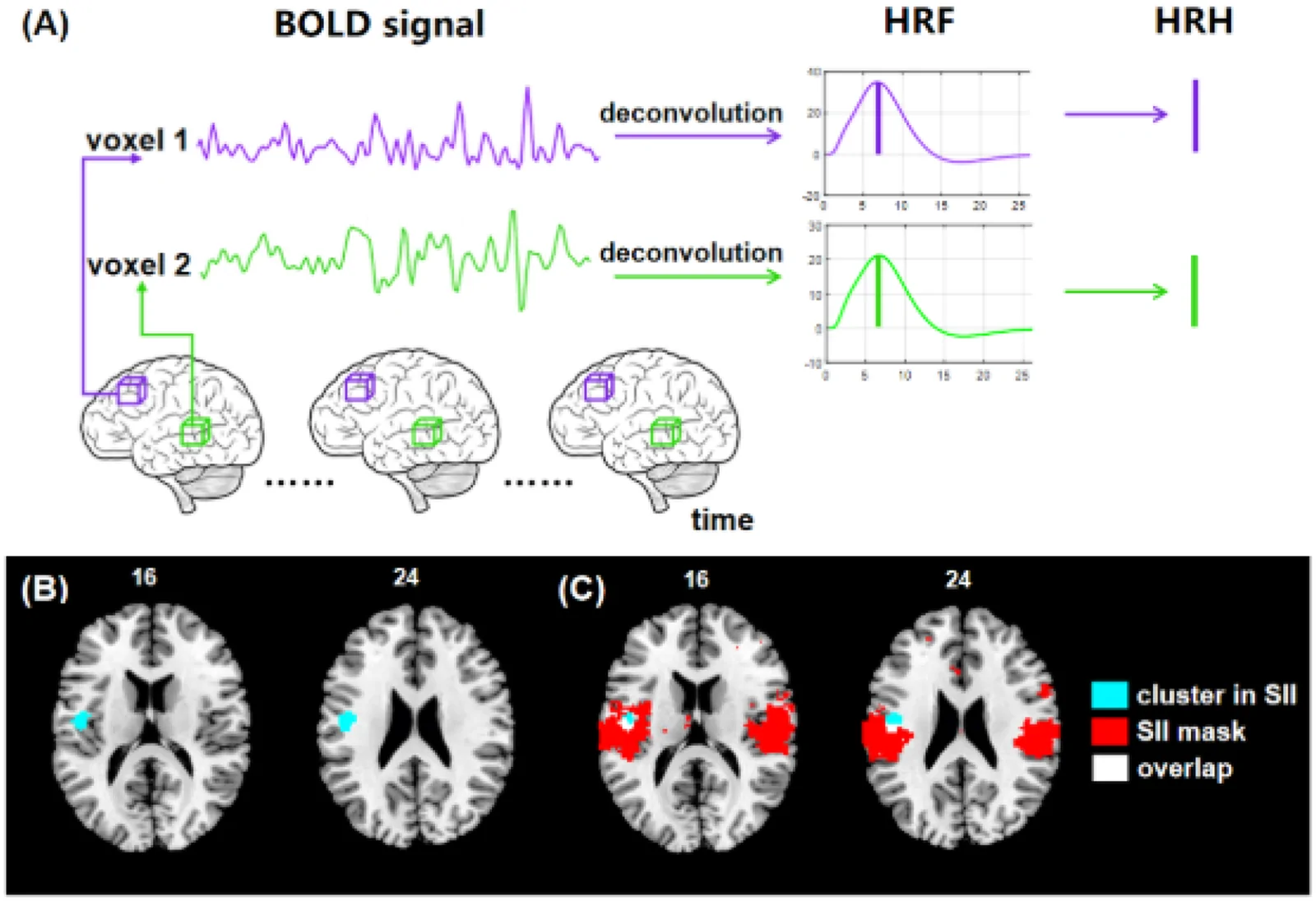
(A) Hemodynamic response function (HRF) and response height (HRH); (B) A cluster in the left SII showed HRH in negative correlation with the PSQI score; (C) The left SII cluster (cyan) highlighted together with a mask of SII (red), identified from Neurosynth search with the key word “secondary somatosensory” and meta-analyses of 150+ papers; the majority of the voxels of the cluster are located within the SII mask. SII: secondary somatosensory cortex.

**Figure 2:**
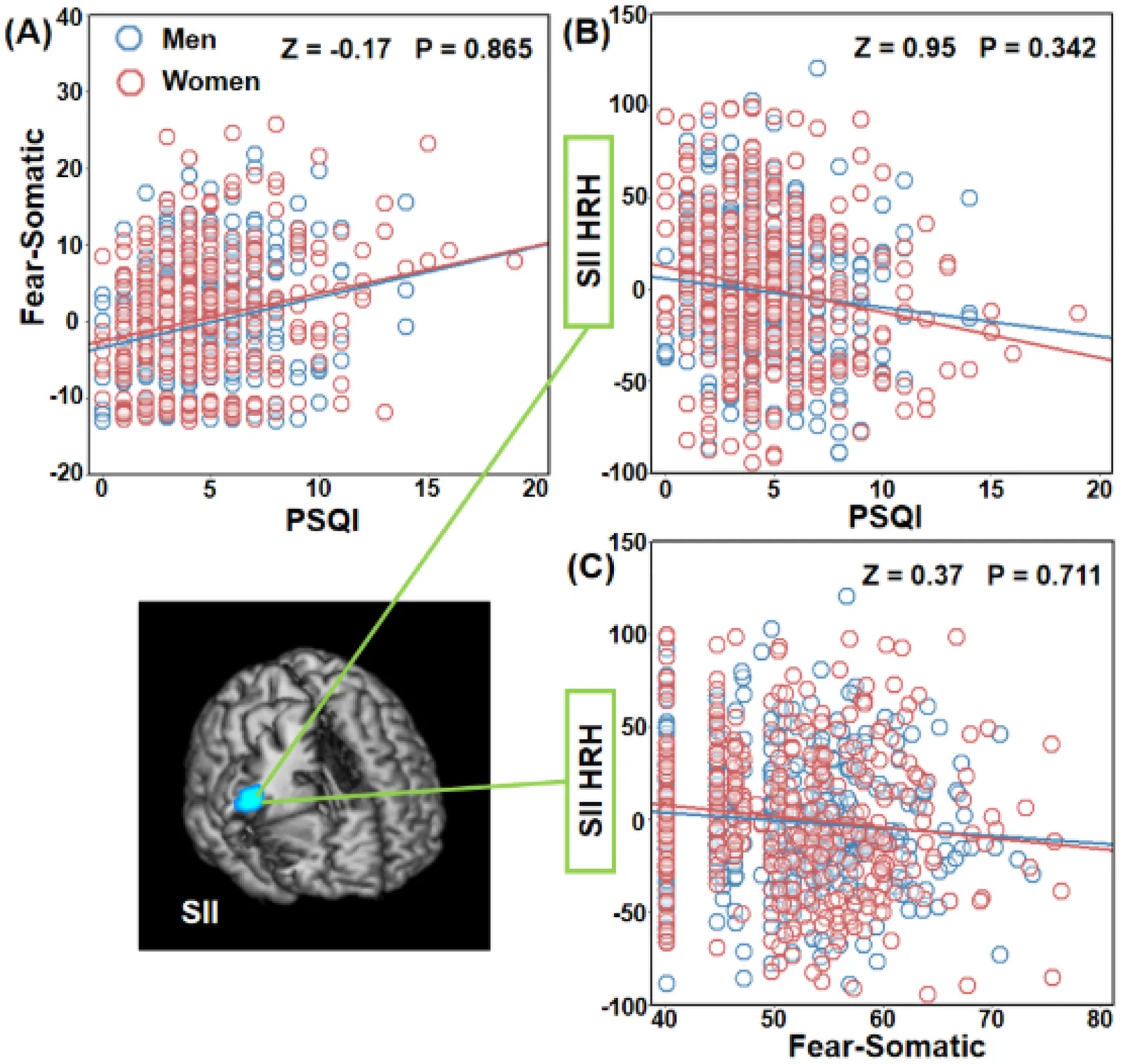
(A) Scatter plots and linear regression of Fear-Somatic vs. PSQI score in men (blue) and women (red); (B) Linear regression of the SII HRH vs. PSQI score and (C) vs. Fear-Somatic score. The *Z* and *p* values reflect the tests of slope differences in the regression between men and women. Note that the residuals are plotted here with age accounted for in all regressions. SII: secondary somatosensory cortex; HRH: hemodynamic response height.

**Figure 3:**
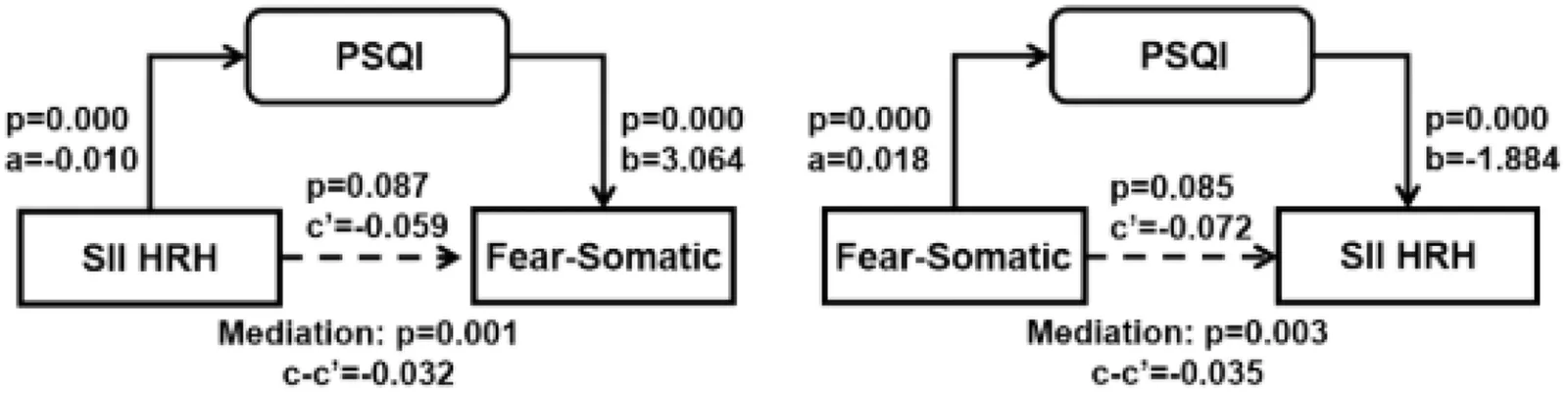
Mediation analyses: PSQI score completely mediated the correlation between SII HRH and Fear-Somatic scores in both directions.

**Table 1:** Demographics and clinical measures of the participants

Characteristic	Men (n = 362)	Women (n = 373)	t	p value*
Age, years	27.6 ± 3.6	29.3 ± 3.7	−6.18	0.000
Education, years	14.9 ± 1.7	15.2 ± 1.7	−1.90	0.058
PSQI score	4.5 ± 2.5	4.7 ± 2.9	−0.99	0.322^
Fear-Somatic	51.4 ± 7.8	51.8 ± 8.2	−1.28	0.202^

Note: Values are mean ± SD;

* two-sample t test (ŵith age as covariate).

PSQI: Pittsburgh Sleep Quality Index.

**Table 2:** Mediation statistics of PSQI, Fear-Somatic scores, and SII HRH

	Path a	Path b	Path c	Path c'	Mediation	Proportion
	(X→M)	(M→Y)	(X→Y)	(X→Y)	Path (c-c')	(c-c')/c, %
Model 1: X (PSQI)→Y (SII HRH) mediated by M (Fear-Somatic)						
*β*	3.190	−0.072	−2.114	−1.884	−0.230	10.88%
*p*	0.000	0.085	0.000	0.000	0.096	
*Model 2:* X (PSQI)→Y (Fear-Somatic) mediated by M (SII HRH)						
*β*	−2.114	−0.059	3.190	3.064	0.126	35.30%
*p*	0.000	0.087	0.000	0.000	0.121	
Model 3: X (SII HRH)→Y (PSQI) mediated by M (Fear-Somatic)						
*β*	−0.092	0.018	−0.010	−0.009	−0.002	20.00%
*p*	0.008	0.000	0.000	0.000	0.017	
**Model 4: X (SII HRH)**→**Y(Fear-Somatic) mediated by M (PSQI)**						
*β*	−0.010	3.064	−0.092	−0.059	−0.032	**34.78%**
*p*	0.000	0.000	0.008	**0.087**	**0.001**	
**Model 5: X (Fear-Somatic)**→**Y(SII HRH) mediated by M (PSQI)**						
*β*	0.018	−1.884	−0.107	−0.072	−0.035	**32.71%**
*p*	0.000	0.000	0.008	**0.085**	**0.003**	
Model 6: X (Fear-Somatic)→Y(PSQI) mediated by M (SII HRH)						
*β*	−0.107	−0.009	0.018	0.018	0.001	5.56%
*p*	0.008	0.000	0.000	0.000	0.037	

Note: significant models were highlighted by bold font.

## Data Availability

All data needed to evaluate the conclusions in the paper are shown in the paper and the supplement. All raw data supporting the analyses and findings of this study are available from the Human Connectome Project, at https://www.humanconnectome.org/.
